# Validation of reference genes for the normalization of RT-qPCR gene expression in *Acanthamoeba* spp.

**DOI:** 10.1038/s41598-020-67035-0

**Published:** 2020-06-25

**Authors:** Martina Köhsler, David Leitsch, Norbert Müller, Julia Walochnik

**Affiliations:** 1 Institute of Specific Prophylaxis und Tropical Medicine, Center for Pathophysiology, Infectiology and Immunology, Medical University of Vienna, Vienna, Austria; 2 Institute of Parasitology, Vetsuisse Faculty, University of Bern, Bern, Switzerland

**Keywords:** Microbiology, Molecular biology, Diseases, Medical research

## Abstract

Acanthamoebae are potentially pathogenic organisms, with a highly unique, yet still insufficiently investigated metabolism. Many open questions can be addressed by gene expression studies, however, for *Acanthamoeba* reliable standards have not yet been established. In this study, suitable reference genes (RGs) for RT-qPCR in *Acanthamoeba* were comprehensively evaluated, comparing different *Acanthamoeba* strains and employing four different algorithms (NormFinder, GeNorm, BestKeeper and RefFinder). Expression stability was assessed under various conditions and the potentials of the most promising RGs for accurate normalization of target genes were evaluated. Expression stability of RGs varied depending on conditions and employed algorithms, however, the genes for the 18S rRNA and the hypoxanthine phosphoribosyl transferase seem to be widely suitable RGs. Normalization with a combination of two carefully chosen RGs resulted in reliable expression data for target genes, while normalization with unsuitable RGs led to significant misinterpretation of expression profiles. Thus, a careful evaluation of RGs prior to expression studies is essential.

## Introduction

Acanthamoebae are ubiquitous, free-living amoebae that are able to inhabit a wide range of environments. However, under certain circumstances they can act as facultative pathogens being the causative agents of *Acanthamoeba* keratitis, an often seriously progressing keratitis, occurring predominantly in contact lens wearers. Furthermore, they can cause several disseminating infections, mostly in immunocompromised individuals, potentially resulting in granulomatous amoebic encephalitis (GAE). Treatment of these infections still poses a problem, since specific amoebicidal agents are not available. Acanthamoebae can also act as vehicles for bacteria, including important human pathogens such as legionellae that resist degradation after phagocytosis. Under unfavorable conditions, the motile trophozoite stage of *Acanthamoeba* can develop into highly resistant cysts, a strategy which is also employed as response to treatment in the course of disease, often leading to recurrent infections^1^.

Acanthamoebae can easily be cultured in the laboratory and make an attractive model organism for cellular differentiation, evolutionary studies and studies on microbial interactions. Furthermore, insights in the complex genome of *Acanthamoeba* revealed that these organisms possess quite unique metabolic strategies and a highly evolved cellular repertoire, arisen from extensive lateral gene transfer^2^. Many aspects in *Acanthamoeba* cell biology are still far from being fully elucidated. The exact mechanisms that are at work during the complex encystment process, pathogenesis in the course of *Acanthamoeba* infections and what determines the potential pathogenicity of *Acanthamoeba* strains are just a few examples, where further research is clearly required.

The elucidation of these complex processes begins by investigating the expression of genes of interest. An extremely potential, yet relatively simple technique is reverse transcription-based, quantitative real-time PCR (RT-qPCR), which has become the method of choice for the quantification of RNA. The RT-qPCR is a highly sensitive and very accurate technique. However, in order to obtain reliable and reproducible results, several important aspects have to be considered. Of course, initial steps such as RNA extraction and the respective RNA quality, reverse transcription and general considerations concerning RT-qPCR and PCR efficiencies have to be carefully evaluated.

However, one of the most crucial steps in RT-qPCR is the choice of a proper normalization method. The best and most common method utilized for the analysis of relative changes in the mRNA expression of target genes is the parallel quantification of standard reference genes (RGs), which ideally are constitutively expressed housekeeping genes, required for the maintenance of basic cellular functions at all time^3^. This strategy eliminates variation introduced by pre-PCR and PCR processing. However, it has been shown that no RG is truly stably expressed over all kinds of culture conditions or among different cell types. This might be explained by the fact that housekeeping genes also participate in other functions than basal cell metabolism^4^. Thus, it is recommended to always use at least two RGs, in order to avoid substantial errors^5,6^. Furthermore, a careful evaluation of the employed RGs prior to large experiments is advisable in addition to following guidelines established for quantitative real-time PCR^7^.

Most often used RGs, in particular during the first years of RT-qPCR, have been the 18S rRNA gene, β-actin (ACTB) and GAPDH, and while accurate and useful in some studies, depending on the experimental setup, the use of these RGs might also lead to significant falsification of expression data^8–10^. In most studies on *Acanthamoeba* RNA expression, these commonly used RGS were employed^11–13^ although no reliable information about their (constitutive) expression pattern under different growth condition was available.

For future studies investigating different *Acanthamoeba* strains under different conditions, reliable RGs are essential. The aim of this study was to evaluate optimal RGs for the normalization of *Acanthamoeba* RT-qPCR. Initially, in order to eliminate inappropriate candidates, selected RGs were tested with four different *Acanthamoeba* strains under two different conditions – namely during the logarithmic growth phase, when amoebae are actively dividing, and during the stationary phase, when nutrients get scarce and amoebae stop dividing, usually resulting in encystment in the long run. The applicability of the tested RGs was evaluated employing NormFinder^14^, geNorm^15^, BestKeeper^16^ and RefFinder^17^, different algorithms developed to assess the stability of a normalizing gene. Additionally, geNorm enables an evaluation, how many RGs are optimally required for an accurate normalization.

Based on these initial findings the most promising RGs were additionally tested under different, more challenging conditions. Amoebae were subjected to induction of encystment, oxidative stress induced by hydrogen peroxide and heat shock, followed by RT-qPCRs with the selected RGs and a target gene, susceptible for a change of expression due to these conditions, in order to evaluate the accuracy of normalization with the selected RGs.

## Results

### Selection of candidate RGs

Of the 12 chosen potential RGs, 11 were selected to be tested regarding their suitability for RT-qPCR-based gene expression in LOG and STAT *Acanthamoeba* cells. The initial selection was based on RT-qPCR efficiency and dispersion of quantification cycle (Cq) values. The Cq value represents the number of cycles required for the fluorescent signal of a sample to cross the threshold line and is inversely proportional to the amount of target nucleic acid in the sample. In this investigation, only TUB was excluded from the experiment because several primer pairs applicable in conventional PCR yielded very low efficiencies in RT-qPCR (Cq values >35) (not shown). RGs including corresponding GenBank accession numbers, primer sequences, and amplicon lengths as well as annealing temperatures and calculated PCR efficiencies of the respective RT-qPCRs are listed in Table 1.

**Table 1:** Selected RGs, primer sequences, amplicon characteristics and source or GenBank accession numbers of published *Acanthamoeba* sequences on which primer design was based.

Primer	Primer sequence (5′-3′)	Amplicon length (bp)	Average Tm(°C)	Amlification efficiency (%)	Source (GenBank accession no.)
18SQV	F: CCCAGATCGTTTACCGTGAA	179	58.4	96.4	Qvarnstrom *et al*. 2004
	R: TAAATATTAATGCCCCCAACTATCC		60.9		
18S900	F: GCCCAGATCGTTTACCGTGA	147	60.5	104.1	18S rRNA gene T4
	R: CATTACCCTAGTCCTCGCG		59.5		
ACT	F: ACCATCGGCAACGAGCG	268	57.6	103.6	XM_004340051
	R: GAGTACTTGCGCTCGGG		57.6		
HPRT	F: GGAGCGGATCGTTCTCTG	201	58.4	100.5	XM_004337011
	R: ATCTTGGCGTCGACGTGC		58.4		
PBGD	F: CCACTCTCTGAAGGACCT	254	56.3	100.2	XM_004340479
	R: GAGTCCAACTTGCGCAGT		56.3		
G6PD	F: CTCACTCTGGACGATCTC	167	56.3	93.3	XM_004338803
	R: GAGGATGAATGGAGTGCC		56.3		
GAPDH	F: CAGCAAACACCACTCTCACG	171	60.5	92.9	U85500
	R: GTACTTGAGGTTGTACACCAT		57.5		
TBP	F: GGATTACTCTACCGAGCTTG	202	58.4	100.4	XM_004338406
	R: GCTGTCCATATCGTCGCTG		59.5		
RasC	F: GCAAATCCTCAGGGTAAAGG	256	58.4	92.5	XM_004344539
	R: CCTTGTTTCCCTTACCCTTG		58.4		
GLP	F: CAACAACACGCTCTCGATC	192	57.5	102.9	XM_004347066
	R: GAAGAGGAACTGCTGCTTG		57.5		
TPI	F: CAGCTCTTCACACCTTCAC	245	57.5	104.9	XM_004335612
	R: GAGCTTGTGTTCGGCGTG		58.4		
TUB	F:CCAGAAGATCACTGCCAC	195	56.3	48.9	DQ099494
	R:GTACTGCTGGTACTCCTG		56.3		

### Expression levels of selected candidate RGs

**Figure 1:**
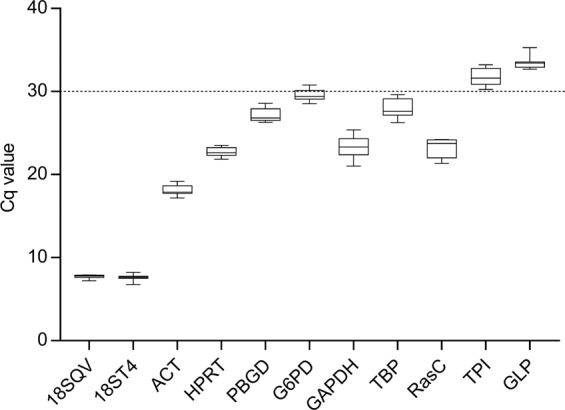
Box plot comparison of mean Cq values after efficiency correction for selected RGs obtained by RT-qPCR analyses of LOG and STAT *Acanthamoeba* cells of all investigated strains.

In order to determine the dispersion of Cq values of the selected candidate RGs under different physiological conditions representing different cell cycle stages, initial runs were performed with cDNA from LOG and STAT *Acanthamoeba* cells of four strains. A boxplot comparison of mean Cq values of LOG and STAT cultures for all investigated strains after efficiency correction of all investigated candidate RGs is shown in Fig. 1. In Table 2 mean raw Cq values, standard deviations (STD), Cq values after efficiency correction and the coefficient of variation are shown.

**Table 2:** Average Cq values, standard deviation (STD), efficiency corrected Cq values and coefficient of variation (CV) of studied RGs for LOG and STAT *Acanthamoeba* cells of all investigated strains.

Gene	Ø Cq LOG/STAT	STD LOG/STAT	Ø Cq eff. corr.	CV %
18SQV	7.9	0.21	7.7	2.7
18ST4	7.4	0.35	7.6	4.7
ACT	17.7	0.58	18.2	3.3
HPRT	22.6	0.53	22.7	2.3
PBGD	27.1	0.76	27.1	2.8
G6PD	30.6	0.69	29.6	2.2
GAPDH	24.6	1.34	23.3	5.4
TBP	27.9	1.10	28.0	3.9
RasC	24.5	1.11	23.2	4.5
TPI	31.1	0.94	31.7	3.1
GLP	32.7	0.71	33.4	2.2

In the RT-qPCR analyses of LOG and STAT cells, the 18S rRNA gene showed rather low average Cq values of 7.4 and 7.9 for primer pairs 18ST4 and 18SQ, respectively. Relatively high transcription levels were also observed for ACT yielding average Cq values of 17.7. Conversely, the majority of RGs (HPRT, RasC, GAPDH, PBGD, TBP) selected for initial testing demonstrated significantly higher Cq values ranging between 20 and >30 before efficiency correction. G6PD, TPI and GLP exhibited Cq values above 30 before efficiency correction, indicating that these genes are expressed at a very low level or that the PCR is working insufficiently. At this point, TPI and GLP with Cq values above 30 were excluded, however, G6PD was kept for further analyses, since efficiency correction resulted in Cq values slightly below 30 and the CV and STD were acceptable.

The comparatively low STDs (Table 2) determined for the 18SQV and HPRT primer-based amplification reactions were indicative for a relatively stable expression/transcription of the respective genes in LOG and STAT cells. Particularly, this latter observation made the 18S rRNA gene (18SQV) and HPRT the most promising candidate RGs for normalization of RT-pPCR-based analyses of gene expression patterns of *Acanthamoeba* LOG and STAT cells.

### Expression stability of the candidate RGs in LOG and STAT *Acanthamoeba* cells

The expression stability of the candidate RGs in LOG and STAT cells was analysed using geNorm, Normfinder, Bestkeeper and RefFinder, respectively. These programs use different algorithms to determine the stability of potential RGs. In particular, Normfinder ranks the set of candidate RGs according to their expression stability with a cut-off value of 0.15, indicating their suitability for normalization of RT-qPCR-based determination of gene expression. Conversely, geNorm calculates the gene expression stability M for an RG as the average pairwise variation of this particular gene with all other RGs simultaneously tested. This calculation is based on the principle that it stepwise excludes the gene with the highest M value. In addition, geNorm involves a cut-off value of 0.15, below which the inclusion of an additional RG is not required. Finally, BestKeeper provides two indicators for the potential stability of an RG, raw standard deviation (BKSTD) of the Cq values on the one hand. On the other hand, this algorithm provides a coefficient of correlation (r) based on Pearson correlation of each of the RGs to the BestKeeper index, as calculated from the geometric mean of the remaining RGs (BK). Generally, RGs with STD values >1 are considered instable and should be sorted out, whereas genes with r values close to 1.0 are considered the most stable genes. RefFinder is a free web-based tool that performs a quick analysis based on the other three algorithms and the comparative CT method starting from single input of raw Ct values only, providing the geomean of the ranking values.

**Figure 2:**
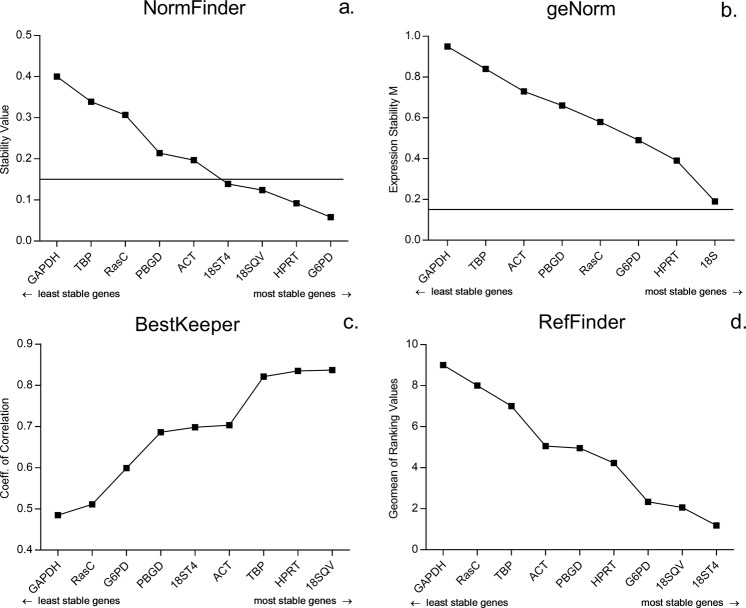
Expression stability of RGs for the normalization of LOG and STAT *Acanthamoeba* cells calculated by different algorithms. RGs are shown in ascending order of expression stability. (**a**) NormFinder stability values, the line indicates the NormFinder cut-off value of 0.15. (**b**) geNorm expression stability M, the line indicates the geNorm cut-off value of 0.15; 18 S = 18 SQV/18ST4. (**c**) BestKeeper coefficient of correlation. (**d**) RefFinder geomean of ranking values.

Output data from all four programs employed for LOG and STAT cells is shown in Fig. 2 and Supplementary Table S1. Additionally, a comprehensive ranking based on all algorithms is shown in Table 3.

**Table 3:** Comprehensive ranking of RGs according to employed algorithms for stability assessment (BK: BestKeeper coefficient of correlation; BKSTD: BestKeeper Standard deviation).

RANK	NormFinder	geNorm	BK	BKSTD	RefFinder	Compr. Ranking
1.	HPRT	18SQV	ACT	18SQV	HPRT	HPRT
2.	18SQV	HPRT	HPRT	18ST4	18SQV	18SQV
3.	G6PD	18ST4	RasC	HPRT	18ST4	18ST4
4.	18ST4	RasC	PBGD	TBP	RasC	RasC
5.	RasC	G6PD	G6PD	G6PD	G6PD/GAPDH	G6PD
6.	GAPDH	GAPDH/TBP	18SQV	GAPDH/RasC		GAPDH
7.	TBP		GAPDH		TBP	TBP
8.	ACT	ACT	TBP	ACT	ACT	ACT
9.	PBGD	PBGD	18ST4	PBGD	PBGD	PBGD

Using NormFinder, the 18S rRNA gene, HPRT and G6PD of *Acanthamoeba* turned out to possess the most stable expression profiles under the different cell culture conditions applied, with expression stabilities below the cut-off value of 0.15. Using geNorm, again the 18S rRNA gene, HPRT and G6PD revealed the most stably expressed genes. However, none of the candidate RGs exhibited an expression stability below a cut-off value of 0.15. This finding suggested a simultaneous use of at least two RGs for normalization. Using BestKeeper, again testing of the 18S rRNA gene and HPRT showed the highest coefficient of correlation with values close to 1. Interestingly, also TBP was among the three best candidates, while this gene was assessed to possess a quite low stability in its expression/transcription profile applying GeNorm and Normfinder. The ranking by refFinder turned out to be concordant with the assessment by the other algorithms used, since the 18S rRNA gene, G6PD and HPRT exhibited the highest expression stabilities.

### Expression stability of the candidate RGs under different conditions

Detailed results for all algorithms employed (NormFinder, geNorm, BestKeeper, RefFinder) are shown in Supplementary Table S1. This table provides exact values from all algorithms for all conditions of all RGs. Comprehensive rankings according to the different algorithms used and referring to the different conditions to which the *Acanthamoeba* cells were exposed are shown in Tables 3 and 4, respectively.

**Table 4:** Comprehensive ranking of RGs based on data obtained from different experimental conditions. (ASAC; all strains, all conditions. LOG/STAT; LOG/STAT cultures. LOG; only LOG cultures. HS; heat shock cultures. OS; oxidative stress with H_2_O_2_. EN; encysting cultures. Neff; strain Neff LOG/STAT/HS/OS/EN data combined).

RANK	ASAC	LOG/STAT	LOG	HS	OS	EN	Neff
1.	HPRT	18SQV	18SQV	HPRT	HPRT	18SQV	HPRT
2.	18SQV	18ST4	G6PD	RasC	18ST4	GAPDH	GAPDH
3.	18ST4	HPRT	18ST4	18SQV/TBP	RasC	18ST4	RasC
4.	G6PD	G6PD	HPRT		GAPDH	HPRT	18SQV
5.	RasC	ACT	ACT	GAPDH	TBP/18SQV	RasC	18ST4
6.	TBP	PBGD	PBGD	G6PD		G6PD	TBP
7.	GAPDH/PBGD	TBP	RasC	18ST4	ACT	TBP	G6PD/PBGD
8.		RasC	TBP	ACT	G6PD	ACT	
9.	ACT	GAPDH	GAPDH	PBGD	PBGD	PBGD	ACT

In the ranking according to the employed algorithms (Table 3), the RGs with the highest stabilites were the 18S rRNA gene and HPRT with NormFinder, geNorm, BKSTD and RefFinder. ACT and PBGD were mostly ranked as the least stable genes. Results based on the coefficient of correlation with BestKeeper (BK) differed from the other algorithms with RasC and ACT among the most stable genes and the 18S gene among the least stable genes, however still ranking HPRT second.

In the ranking based on the comprehensive results of all algorithms considering all different categories of *Acanthameba* cells tested (Table 4), a higher degree of variation became apparent among the top ranked RGs. Here, the 18S rRNA, HPRT and G6PD genes turned out to be comparatively most suitable as references for normalization of gene expression in LOG/STAT, LOG (alone) and ASAC cells. In the case of HS cells, however, RasC and TBP operated similarly well as the 18S rRNA gene and HPRT, respectively. In the case of OS cells after HPRT and 18ST4 also RasC and GAPDH were among the most promising RGs. GAPDH showed also excellent operating characteristics in EN cells, but comparatively exhibited lowest expression stability in LOG/STAT and LOG (alone) cells. In an overall evaluation under all experimental conditions to which *Acanthamoeba* strain Neff was exposed in the present study, HPRT exhibited the most stable expression, followed by GAPDH and RasC that also revealed satisfactory operating characteristics as RG for normalization of the expression patterns in this strain.

**Figure 3:**
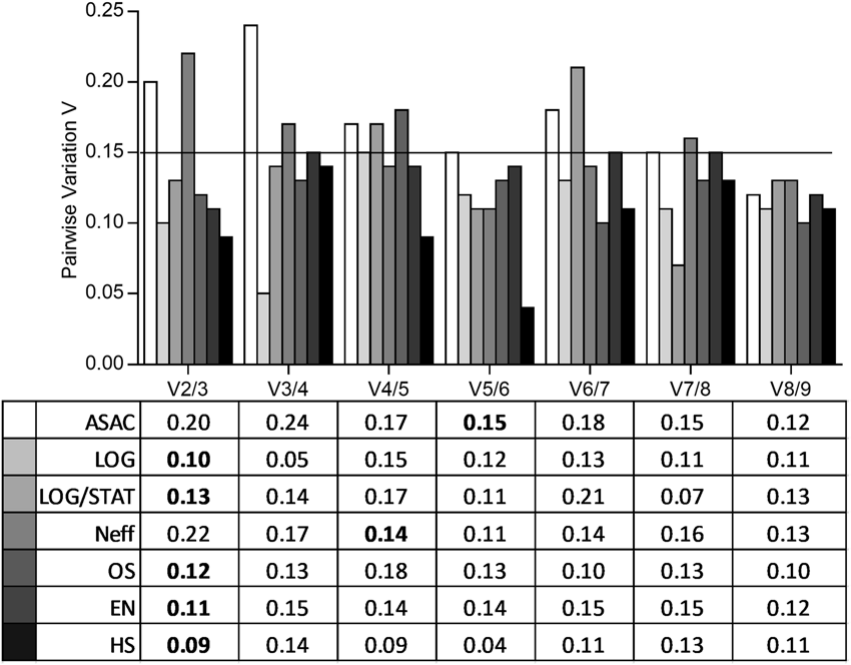
Optimal number of RGs for accurate normalization based on pairwise variation (Vn/Vn + 1). The black line indicates the proposed cut-off value of 0.15, below which the inclusion of an additional RG is not required. (ASAC; all strains, all conditions. LOG/STAT; LOG/STAT cultures. LOG; only LOG cultures. HS; heat shock cultures. OS; oxidative stress with H_2_O_2_. EN; encysting cultures. Neff; strain Neff LOG/STAT/HS/OS/EN data combined).

Gene expression stability values calculated with geNorm were above the cut-off value of 0.15 for all RGs under all experimental conditions (Supplementary Table S1), implying that more than one RG is required for an accurate normalization. Accordingly, pair-wise variation (V) values were calculated to identify the optimal number of RGs required for normalization (Fig. 3). Referring to these V values, the application of at least two RGs is necessary to achieve an accurate normalisation of gene expression patterns in different *Acanthamoeba* strains in their logarythmic phase (LOG), for different strains combining the logarithmic and stationary phase (LOG/STAT), for strain Neff during encystment (EN), after oxidative stress (OS) and after heat shock (HS). For strain Neff exposed to different experimental conditions outlined above, analysis of even four RGs is recommended. In order to obtain reliable data for different *Acanthamoeba* strains exposed to various conditions (ASAC), as much as five RGs would be required to obtain a pair-wise variation value under 0.15 and thus are qualified for an adequate normalization.

### Validation of RGs under different conditions

**Figure 4:**
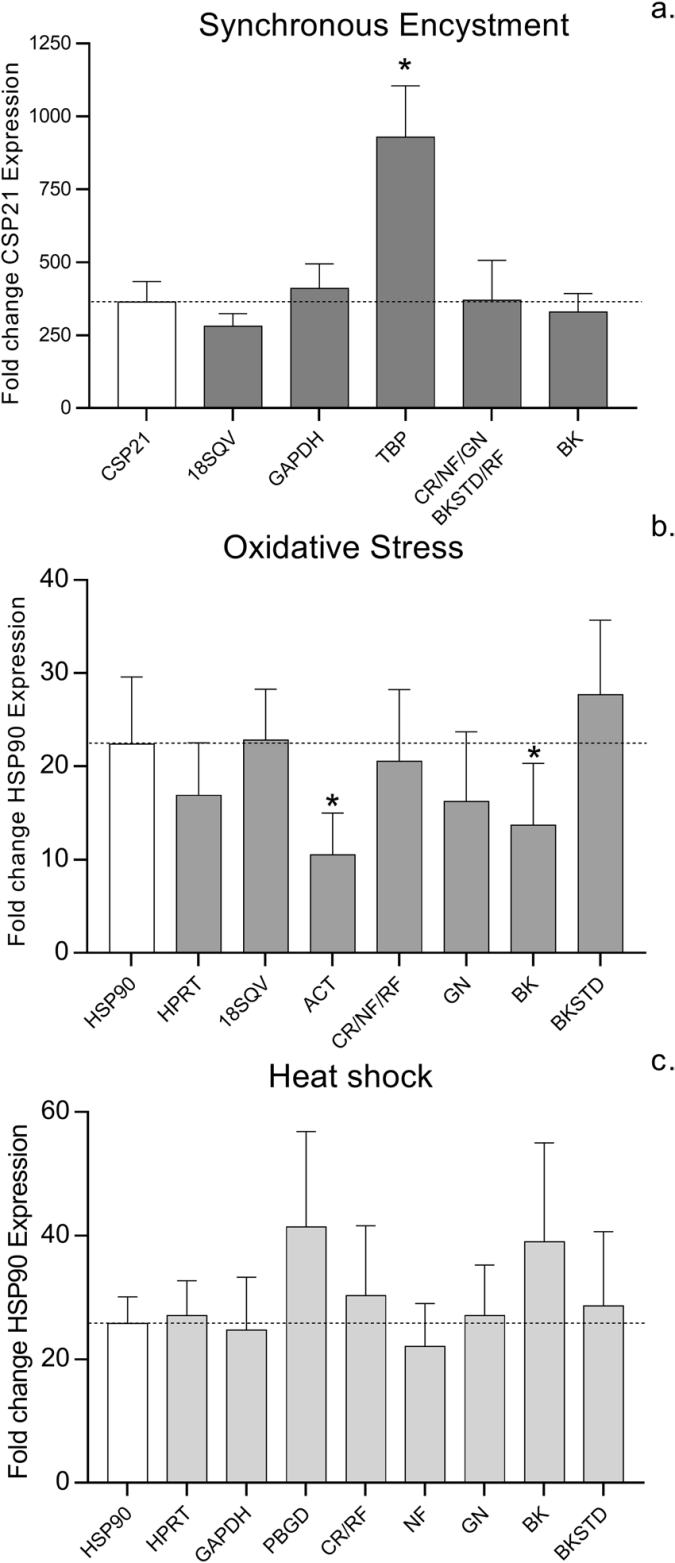
Relative expression of CSP21 during synchronous encystment (**a**) and HSP90 after oxidative stress (**b**) and heat shock (**c**) in *Acanthamoeba castellanii* strain Neff. First column: calculated fold-change of target gene; second column: fold change for best ranked RG (Table 4); third column: RG with most accurate calculated fold change (Supplementary Table S2); fourth column: RG with most inaccurate calculated fold change (Supplementary Table S2). Furthermore, fold changes based on normalization with the two highest ranked RGs for each algorithm are shown (CR; comprehensive ranking. NF; NormFinder. GN; geNorm. BK; BestKeeper coefficient of correlation. BKSTD; BestKeeper Standard Deviation. RF; RefFinder.) Bars represent the means and standard error of three biological replicates. Asterisks indicate a significant difference in fold change of expression compared to calculated fold changes of CSP21 and HSP90 (P < 0.05).

For the validation of the RGs for *Acanthamoeba* cell cultures under different conditions, expression of selected target genes was normalized by independently applying all RGs investigated in this study. Furthermore, we used different combinations of RGs according to their rankings based on different algorithms and pair-wise variaton values described above. Results are shown in Fig. 4a–c. Calculated changes for all RGs and algorithms are provided in Supplementary Table S2.

For the validation of the RGs investigated for encysting cells, CSP21 was chosen as target gene. CSP21 was reported to be repressed in growing cells, but strongly induced during early differentation into the cyst form^18^. The calculation of the RQ of CSP21 for growing cells before induction of encystment and encysting cells resulted in a 364-fold change of CSP21 expression. This value was compared to the calculated changes after 2^-∆∆CT^ normalization with all RGs as well as combinations of the two best ranked genes, as indicated by pair-wise-variation values (Fig. 3), for each algorithm and a comprehensive ranking of all algorithms (CR). Expression of CSP21 based on normalization with the best ranked RG and with the RGs resulting in the most accurate and the most inaccurate calculated fold-changes, and for the combination of two genes according to different algorithms are shown in Fig. 4a. The calculated change for normalization with 18SQV, the best ranked gene, was 279.6-fold, while the most accurate normalization was achieved with GAPDH (410.2-fold). Normalization with TBP resulted in the most inaccurate change of CSP21 expression (928.8-fold), leading to a significantly different expression rate (Fig. 4a). Changes based on normalization with two RGs were 370.4-fold for recommended genes based on Normfinder, geNorm, BestKeeperSTD, Refinder and on the comprehensive ranking (CR) (18SQV, GAPDH) and 329.3-fold based on the coefficient of correlation with BestKeeper (ACT/G6PD).

For the validation of the RGs during oxidative stress in strain Neff, HSP90 was chosen as target gene and calculations were performed as described above. Here, the calculated change of the HSP90 expression after exposure to H_2_O_2_ was 22.4-fold. The best ranked RG was HPRT, resulting in a calculated 16.9-fold change, while the most accurate normalization was achieved with 18SQV, resulting in a 22.8-fold change. Normalization with ACT led to the most inaccurate expression value (10.5-fold). Changes based on two RGs were 20.5-fold based on NormFinder, RefFinder and CR (HPRT/18ST4), 16.2-fold with geNorm (HPRT/RasC), 13.7-fold with BestKeeper (GAPDH, RasC) and 27.7-fold with BestKeeperSTD (18ST4/TBP). Here, normalization based on ACT and BestKeeper coefficient of correlation would lead to a significant misinterpretaton of HSP90 expression (Fig. 4b).

For the validation of the RGs of heat-shocked strain Neff cells, also HSP90 was chosen as target gene. Here, the calculated change in the HSP90 expression after heat shock was 25.8-fold. The best ranked gene was HPRT, resulting in a calculated 27.1-fold change. The most accurate change, however, was calculated for GAPDH with 24.7. Changes calculated based on two RGs were 30.3-fold assessed with RefFinder and CR (HPRT, RasC), 22.1-fold with NormFinder (G6PD, HPRT), 27.1-fold with GeNorm (HPRT, TBP), 39-fold with BestKeeper (ACT, PBGD) and 28.7-fold with BestKeeper STD (18SQV, RasC), respectively. Here, almost all calculations resulted in appropriate normalization results. Only expression based on PBGD, ranked last with all algorithms, except for BestKeeper, resulted in a considerably higher expression of HSP90 (Fig. 4c).

## Discussion

RT-qPCR has been widely used to evaluate gene expression because of its speed and sensitivity. However, a reliable quantification of gene expression mainly depends on an accurate normalization. Common RG choices in a majority of expression studies in various cellular systems were, and still are, GAPDH, ACTB and 18S rRNA^10^. GAPDH and ACTB served as common standards during the early years of Northerm blotting, which was pursued in qPCR^19^. However, it became evident that at least under certain experimental set-ups, normalization of gene expression profiles with these housekeeping genes might result in significant misrepresentation of expression data^8^. It is now well established, that more than one RG should be employed in order to avoid such a bias in the interpretation of respective results^8^. Studies employing RT-qPCR for the evaluation of gene expression in *Acanthamoeba* are still rare and to date most of these studies employed the “usual suspects” for normalization, such as 18S rRNA and GAPDH^12,13,20^.

In this study, we selected eleven potential RGs as candidates to achieve an accurate normalization of RT-qPCR-based testing of gene expression in *Acanthamoeba*. In particular, we investigated genes already used for *Acanthamoeba* (18S rRNA gene, GAPDH) and genes generally considered housekeeping genes and employed as RGs (α-tubulin, ACT, TBP, HPRT, G6PD, TPI). Both, glycogen phosphorylase (GLP), which breaks down glycogen into free glucose, e.g. acting as an intermediate component for cyst wall formation and RasC, involved in cellular signalling, represent genes that have basic cellular functions. In the case of the RasC gene, the suitability as a stable internal control has already been demonstrated^21,22^. Conversely, the α-tubulin-encoding gene was initially excluded for this purpose because several sets of primers targeting this gene did not provide satisfying results concerning both, Cq values (>35) and efficiency (<90%). Furthermore, GLP and TPI were excluded from further experimentation due to their low sensitivity (Ct values >30) in RT-qPCR by testing LOG and STAT cells.

Principally, apart from the fact that optimal RGs are supposed to exhibit a constitutive expression throughout different cellular stages and culture conditions as applied in the present study, another important aspect is that they exhibit an expression level similar to the level(s) of the gene(s) of interest^8^. Since expression values among genes of interest may vary by several orders of magnitude, however, it is impossible to perfectly fulfil this criterion particularly if only one RG has been selected for RT-qPCR-based assessments of gene expression profiles. In most practical applications, however, RGs providing Cq values between 20–30 at appropriate sample dilutions produce reliable and reproducible results. It is important to note that apart from 18S rRNA and ACT genes, these operating characteristics account for the entire panel of *Acanthamoeba* RGs further evaluated in the present study. As far as ACT is concerned still an experimentally acceptable expression level (Cq value: ~18) was evaluated. As expected, however, the 18S rRNA gene showed high expression with Cq values between 7 and 8. In many studies the 18S rRNA shows extremely high expression levels, since ribosomal RNA comprises approximately 80% of the total RNA. Therefore, an initial dilution has already been suggested for accurate normalization^23,24^, which might be reasonable for *Acanthamoeba*. Due to its high abundance and the fact that it is less prone to degradation, the 18S rRNA has been perceived critically in many studies^25,26^. Nonetheless, standard deviations for both 18S primer pairs turned out to be the lowest in almost all experiments of our study, with 18SQV outperforming 18ST4, which was designed for this study to specifically amplify genotype T4, the most abundant genotype, being responsible for the majority of *Acanthamoeba* infections^27^. After our initial experiments with LOG/STAT cultures, the 18S rRNA, HPRT and G6PD were found to be the most stably expressed genes, while clearly GAPDH was the least stable, according to all algorithms. Both NormFinder and geNorm recommend a cut-off value of 0.15 for the stability value and expression stability M, respectively. This was achieved with NormFinder for the 18S rRNA gene, HPRT and G6PD. Using geNorm, however, all RGs were above this cut-off value, indicating that at least two RGs are required for an accurate normalization. The output generated by different algorithms can vary significantly, due to the fact that quite divergent statistical approaches are employed. With LOG/STAT cells, this effect was weak. Expression stabilities according to NormFinder, geNorm and the free web-tool RefFinder, that basically integrates the other algorithms as well as the comparative CT method^28^, were fairly concordant. Only BestKeeper (coefficient of correlation) data output showed minor differences, e.g. as far as the ranking of PBGD and ACT is concerned (Table 3).

The difference in RG expression stability between different algorithms was more pronounced for HS, OS, and encysting cells (Table 4). Again, the 18S rRNA gene and HPRT were among the most stably expressed genes, however, GAPDH which was evaluated to be the least stable gene in LOG/STAT and LOG cells, was generally ranked higher. Also RasC showed higher expression stabilities in HS, OS, and encysting cells as compared to LOG/STAT and LOG cells.

Compared to all other algorithms, ranking according to the BestKeeper coefficient of correlation showed a different output identifying ACT as the most stably expressed gene, RasC third best and both RT-qPCRS targeting the 18S rRNA gene ranking in lower positions (Table 3). Such discrepancies particularly between BestKeeper and other algorithms are common and have been reported previously^29–31^. This might be due to the unique configuration of the BestKeeper algorithm. For example, BestKeeper is not ideal for a comparison of RGs exhibiting very different expression levels. This is the case because respective expression values cannot be correlated parametrically^16^, which might be responsible for the discrepancies in the ranking of the 18S rRNA primers.

Only geNorm provides a method to define the optimal number of RGs for accurate normalization of gene expression by generating the pairwise variation V value (Fig. 3.) V2/3 is supposed to be below the threshold value of 0.15 for two RGs to be sufficient for data normalization; otherwise another RG has to be added. For most experimental conditions applied, two RGs (V2/3 ≤ 0.15) were found to be optimal. However, based on these V values for different conditions in strain Neff and all strains and all conditions analysed together (ASAC) the implementation of four (V4/5 ≤ 0.15) or even five (V5/6 ≤ 0.15) RGs would be recommended. This might partly be explained by the multifactorial character of our investigation. Several independent experiments with different set-ups were taken into account, although all experiments followed a standardized protocol regarding number of cells, identical amounts of RNA for cDNA transcription and identical amounts of cDNA employed in each qPCR. Here, the independent experiments including multiple analytical parameters provided a certain variation in Cq values for all RGs, which might also be attributed to differences in cDNA quality or variabilities in the individual qPCR runs. The necessity to include four or even five RGs in order to obtain reliable results for the comparative determination of cellular expression profiles by qPCR has already been reported previously^15^. However, the necessity to employ more than three RGs should give rise to consider testing different RG candidates in order to save time and reduce cost. As a general rule it is recommended to use at least three RGs in three biological replicates^7,8^.

For the validation of RGs in encysting cells all algorithms, except BestKeeper, suggested the combination of the same two RGs and leading to accurate normalization of CSP 21 (Fig. 4a). Normalization with TBP alone, however, would result in a significantly higher CSP21 fold change than the calculated RQ, indicating that TBP expression is upregulated during the encystment process. This stands in contrast to reports that TBP levels remain unaltered during encystment^32^. Also actin is a poor choice for normalization during encystment, as it resulted in notably lower CSP21 compared to the calculated RQ (Supplementary Table S2). This is in agreement with the fact that the encystment process of *Acanthamoeba* is accompanied by cytoskeletal rearrangements, and actin synthesis has been demonstrated to be involved in this process, with a slight repression of transcription during early encystment and a regulation of actin at the translational level^33^.

Although varying combinations of RGs were indicated by different algorithms for the normalization of HSP90 after heat shock and oxidative stress, all, with the exception of RGs based on BK coefficient of variation, resulted in rather accurate expression levels with similar fold-changes compared to the calculated fold-change based on calculated RQ. For both conditions, ACT and PBGD appear to be unsuitable as RGs resulting in significantly different expression rates. An explanation for the poor performance after heat shock and oxidative stress, might be that actin has been reported to interact with HSP90^34^ while PBGD, has been reported to be involved in the stress response in *Aspergillus*, hence affecting its expression^35^. While normalization with GAPDH yielded accurate fold changes after heat shock, it would be an unsuitable RG under oxidative stress conditions with diverging expression levels. The influence of oxidative stress on GAPDH has been demonstrated in mice^36^, and also in plants certain stress conditions have been reported to affect the expression level of GAPDH^8^.

As mentioned earlier, the RG set investigated in this study exhibited a wide range in Cq values, ranging from 8 to almost 30, for which the BestKeeper algorithm might not be ideal, at least when based on the coefficient of correlation. This was corroborated by the results of RG validation, where normalization based on BK coefficient of correlation gave the most inaccurate expression levels. However, normalization employing the recommended RGs based on the comprehensive ranking, which naturally was almost identical with ranking according to RefFinder, led to very accurate expression levels, indicating that a combination of several different algorithms, combining results from different mathematical approaches, ensure the most adequate results.

Altogether, HPRT and the 18S rRNA gene appear to be a safe choice for all kinds of experiments in *Acanthamoeba* spp. Despite all possible factors that might argue against the use of the 18S rRNA gene, it performed convincingly in our study. HPRT, with stable expression throughout all experiments and moderate expression levels (Ct: ~23) in our study, has been shown to be among the most stable RGs in diverse other studies evaluating RG stability^37,38^. However, G6PD, RasC and GAPDH might be sound options as well. G6PD was among the most stable genes in experiments with different *Acanthamoeba* strains, indicating that expression levels between strains are similar, while G6PD was clearly more affected by a change of conditions. GAPDH, however, appears to exhibit different expression rates in different strains, while being less affected by a change of conditions within just one strain, as observed when only *A. castellanii* strain Neff was investigated.

In conclusion, we found that while the 18S rRNA gene and HPRT appear to be promising RGs for the normalization of gene expression analyses in different *Acanthamoeba* strains exposed to different experimental conditions, a careful evaluation of RG expression stability prior to the actual experiments is clearly preferable. Furthermore, our results indicate that normalization with only one RG can lead to a biased data output, while a combination of at least two RGs based on the output of several algorithms clearly resulted in more accurate expression rate determinations.

## Materials and methods

### Strains and culture conditions

Four *Acanthamoeba* strains, all representing genotype T4 were used in this study, *A. castellanii* strain Neff (ATCC 30010), isolated from soil in the year 1957, and three strains isolated from corneal scrapings of patients, who had developed an *Acanthamoeba* keratitis, namely strain 1BU, isolated in the year 2000^39^, strain SPA08, isolated in the year 2008^40^ and strain STR16 (isolated in 2016). All strains were grown at 25 °C in 20 ml sterile filtrated proteose peptone yeast extract-glucose medium (PYG) in 75-cm^2^ tissue culture flasks with weekly medium changes.

### Selection of candidate RGs

Apart from primers AcantF900 and AcantR1100 (primers  18SQV), which have been established for *Acanthamoeba* detection^41^, all primers were designed based on published *Acanthamoeba* sequences from GenBank. Referring to general recommendations from other publications and sequence availability, eleven candidate RGs were chosen for primer design. The genes selected were the 18S rRNA gene (18ST4), actin-1 (ACT), hypoxanthine-guanine phosphoribosyltransferase (HPRT), porphobilinogen-deaminase (PBGD), glucose-6-phosphate dehydrogenase (G6PD), glycerol-3-phosphate dehydrogenase (GAPDH), TATA-binding-protein (TBP), RAS GTPase (RasC), glycogen phosphorylase (GLP), triosephosphate isomerase (TPI) and β-tubulin (TUB). For most RGs at least two primer pairs were designed and initially tested in conventional PCR with a standard program using genomic DNA and RNA from strain Neff. Subsequently, a three-fold serial dilution from Neff DNA and cDNA was used to construct standard curves to determine the PCR amplification efficiencies (E) for each primer pair. E was determined based on the slopes of the standard curves: E(%) = (10^1/slope^ − 1) × 100%. The primer pair with better performance in conventional PCR and better efficiency in RT-qPCR was chosen for further experiments. The selected RGs and respective primer pairs chosen for further experiments are shown in Table 1. TUB primers were excluded after initial tests.

### RNA isolation and cDNA synthesis

Initial experiments were undertaken with amoebae in the logarithmic growth phase (LOG cells) and in the stationary growth phase (STAT cells), respectively. For logarithmic and stationary cells, 10 ml amoebae-containing medium from culture flasks were transferred into fresh flasks and filled up with fresh medium to 20 ml and grown for another 48 h for LOG cells and for ten days for STAT cells before harvesting. For all preparations, amoebae were counted using a Fuchs-Rosenthal haemocytometer. 3 × 10^6^ amoebae were harvested by centrifugation (700 g/10′) and washed twice with PBS. RNA was isolated using the GeneJET RNA Purification Kit (Thermo Fisher Scientific) following the manufacturer’s instructions. RNA was treated with DNase I (Roche) to remove contaminating genomic DNA. The RNA concentration and purity were determined using a NanoDrop spectrophotometer ND1000 (NanoDrop Technologies). Only samples with a 260/280 ratio between 1.8 and 2.0 were used for subsequent analyses. RNA integrity was assessed by 1% agarose gel electrophoresis. For cDNA synthesis, the amount of total RNA was standardized to 1 µg per reaction. First-strand cDNA was synthesized using the Maxima First Strand cDNA Synthesis Kit for RT-qPCR (Thermo Fisher Scientific). Then, cDNA was quantified using the ND1000. All cDNA samples were diluted to 10 ng/µl using DEPC-treated water and stored at −80 °C for further processing.

### Quantitative real-time PCR

RT-qPCR was performed in a CFX96 thermocycler (Bio-Rad) using Takyon No Rox SYBR Master Mix dTTP Blue (Eurogentec) in 96-well white PCR plates sealed with Absolute qPCR seals (Bio-Rad). The reaction mixture (15 µl per reaction) contained 7.5 µl 2xMasterMix, 200 nM of each primer and 70 ng cDNA (7 µl of 10 ng/µl). No-template reactions were used as negative controls. The RT-qPCR temperature profile included an initial denaturation step at 95 °C for 3 min, followed by 45 cycles of 15 s at 95 °C, 15 s at 55 °C and 15 s at 72 °C. A melting curve was performed at the end of the RT-qPCR run by stepwise (0.5 °C per 5 s) increasing the temperature from 65 °C to 95 °C. All experiments were carried out in at least three independent set ups. For each cDNA batch, three independent RT-qPCR runs were performed. Additionally, RNA samples were included in the first RT-qPCR run with 18SQV primers in order to detect the presence of contaminating gDNA.

### Expression data and RG stability

For the determination of Cq (quantification cycle) values, the same baseline threshold was set for all experiments in the single threshold mode of the CFX Manager Software (Bio-Rad). Efficiency-corrected Cq values were calculated by the formula CqE = Cq*(log(E)/log(2)). E stands for the PCR efficiency, which was determined earlier for all primer pairs (Table 1). The coefficient of variation (CV) was calculated as CV = σ/µ where σ corresponds to the standard deviation of the Cq values of a candidate RG and µ corresponds to the mean Cq value for the same gene. To determine the stability of expression for the RGs investigated, the software tools geNorm (https://genorm.cmgg.be), NormFinder (https://moma.dk/normfinder-software), BestKeeper (https://www.gene-quantification.de/bestkeeper.html) and the web-based tool RefFinder (https://heartcure.com.au) were employed. For geNorm and Normfinder raw Cq values had to be transformed into relative quantities (RQ), using the formula RQ = E^−∆Cq^. ∆Cq was calculated using the formula ∆Cq = Cqmin − Cqsample, with Cqsample being the raw Cq value for each gene, and Cqmin being the minimal raw Ct value (the sample with the highest relative quantity) over a range of samples. In contrast, for BestKeeper and RefFinder the raw non-transformed Cq values were used.

### Assessment of RG expression stability under different conditions

In order to evaluate the stability of the expression for the selected RGs, amoebae of strain NEFF in the logarithmic growth phase were exposed to more challenging conditions, namely synchronous encystment (SE), heat shock (HS) and oxidative stress (OS). For generation of SE cells logarithmically growing amoebae were washed with PBS and then cultured in encystment medium (95 mM NaCl, 5 mM KCl, 8 mM MgSO_4_, 0.4 mM CaCl_2_, 1 mM NaHCO_3_ and 20 mM Tris-HCl, PH 9.0) for 15 h at RT prior to harvesting. For generation of HS cells, growth temperature was shifted for 1 h from RT to 37 °C, and for generation of OS cells, amoebae were exposed to 1 mM hydrogen peroxide (H_2_O_2_) for 1 h prior to harvesting of cells for RNA isolation. Again, 3 × 10^6^ amoebae (SE, HS, OS) as well as untreated amoebae in their logarithmic growth phase were harvested for RNA isolation and subsequent cDNA synthesis (see above). All cDNA samples were diluted to 10 ng/µl using DEPC(diethyl pyrocarbonate)-treated water and stored at −80 °C. All experiments were done in three biological replicates. RT-qPCRs were run in triplicates with all candidate RGs, with the exemption of TPI and GLP. For SE cells, a cyst-specific protein 21 (CSP21) gene segment was chosen as target sequence, using primer pair CSP21fw(5′-AGGAGTGGAACATGAGGAG-3′) and CSP21rev(5′-GCCTTCTTCCTTACACATGC-3′) for the amplification reaction. For HS and OS cells, a heat shock protein 90 (HSP90) gene segment was chosen as target sequence, employing primers HSP90fw(5′-GGTCATCAAGGACATCCTC-3′) and HSP90rev(5′-GTAGGTCTG-CATCGAGCTG-3′). All primers were designed based on published *Acanthamoeba* sequences from GenBank. The expression stabilities of the RGs in HS, OS, SE cells of strain Neff were again individually assessed employing geNorm, NormFinder, BestKeeper and RefFinder. The same analyses were performed for all conditions for strain Neff combined (Neff), for LOG cultures alone (LOG) and additionally for all strains and all conditions together combining all experiments (ASAC). Again, raw Cq values were used for BestKeeper and RefFinder. Conversely, geNorm and NormFinder RQs were calculated as described above. Pair-wise variation (V) was calculated with geNorm to identify the optimal number of RGs required for an accurate normalization for conditions tested. Here, the cut-off V value is 0.15, below which the addition of another internal control gene does not result in a significant improvement of normalization.

### Validation of RGs

The relative change in gene expression of the target genes (CSP21, HSP90) was analysed using the 2^−∆∆CT^ method, employing all RGs separately for normalization. Additionally, based on pair-wise variation, expression patterns of target genes were normalized with the two best-ranked RGs applying each algorithm (Table 3). The relative change in expression upon normalization was compared to the calculated RQ of each target gene based on the formula RQ = E^−∆Cq^, by comparing Cq values of logarithmically grown Neff cells with ES, HS and OS cells. This calculation was based on the fact that each RT-qPCR was done with the same amount of cDNA (10 ng/1 µl per reaction). Student’s t-test was performed to compare calculated fold-changes and fold-change based on normalization with RGs with significance reported for p < 0.05.

## Supplementary information

Supplementary information.
