# Supplementary material for: Validation of reference genes for the normalization of RT-qPCR gene expression in Acanthamoeba spp
Source: Sci Rep. 2020 Jun 25;10:10362. doi: 10.1038/s41598-020-67035-0 (PMC7316857; doi:10.1038/s41598-020-67035-0)
Supplement: Supplementary file 1 — Supplementary information. [file 41598_2020_67035_MOESM1_ESM.docx]

Validation of reference genes for the normalization of RT-qPCR gene expression in *Acanthamoeba* spp.

Martina Köhsler^1^, David Leitsch^1^, Norbert Müller^2^, Julia Walochnik^1*^
^1)^ Institute of Specific Prophylaxis und Tropical Medicine, Center for Pathophysiology, Infectiology and Immunology, Medical University of Vienna, Austria
^2)^ Institute of Parasitology, Vetsuisse Faculty, University of Bern, Switzerland

*correspondence: julia.walochnik@meduniwien.ac.at

**Supplementary Table S1**. Expression stability values for all RGs calculated with all algorithms and all conditions tested. (ASAC; all strains, all conditions. LOG/STAT; LOG/STAT cultures. LOG; only LOG cultures. HS; heat shock cultures. OS; oxidative stress with H_2_O_2_. EN; encysting cultures. Neff; strain Neff LOG/STAT/HS/OS/EN data combined.)

| NormFinder | | | | | | | | | | | | | | | |
| --- | --- | --- | --- | --- | --- | --- | --- | --- | --- | --- | --- | --- | --- | --- | --- |
| Gene | Stability value | | | | | | | Ranking | | | | | | | |
|  | ASAC | log/ stat | log | HS | OS | EN | Neff |  | ASAC | log/stat | log | HS | OS | EN | Neff |
| 18SQV | 0.38 | 0.12 | 0.06 | 0.21 | 0.18 | 0.33 | 0.33 | 18SQV | 2 | 3 | 1 | 5 | 4 | 2 | 2 |
| 18ST4 | 0.50 | 0.14 | 0.09 | 0.25 | 0.14 | 0.44 | 0.42 | 18ST4 | 5 | 4 | 3 | 7 | 1 | 4 | 5 |
| ACT | 0.76 | 0.20 | 0.41 | 0.41 | 0.31 | 0.68 | 0.73 | ACT | 9 | 5 | 5 | 8 | 6 | 9 | 9 |
| HPRT | 0.34 | 0.09 | 0.13 | 0.08 | 0.16 | 0.41 | 0.31 | HPRT | 1 | 2 | 4 | 2 | 2 | 3 | 1 |
| PBGD | 0.60 | 0.21 | 0.48 | 0.44 | 0.45 | 0.62 | 0.66 | PBGD | 7 | 6 | 6 | 9 | 9 | 7 | 8 |
| G6PD | 0.44 | 0.06 | 0.06 | 0.06 | 0.43 | 0.56 | 0.59 | G6PD | 3 | 1 | 1 | 1 | 8 | 6 | 7 |
| GAPDH | 0.63 | 0.40 | 0.72 | 0.24 | 0.22 | 0.32 | 0.34 | GAPDH | 8 | 9 | 9 | 6 | 5 | 1 | 3 |
| TBP | 0.53 | 0.34 | 0.66 | 0.10 | 0.18 | 0.63 | 0.54 | TBP | 6 | 8 | 8 | 4 | 3 | 8 | 6 |
| RasC | 0.46 | 0.31 | 0.59 | 0.09 | 0.36 | 0.53 | 0.35 | RasC | 4 | 7 | 7 | 3 | 7 | 5 | 4 |
| geNorm | | | | | | | | | | | | | | | |
| Gene | Expression stability M | | | | | | | Ranking | | | | | | | |
|  | ASAC | log/ stat | log | HS | OS | EN | Neff |  | ASAC | log/stat | log | HS | OS | EN | Neff |
| 18SQV | 0.51 | 0.19 | 0.21 | 0.37 | 0.79 | 0.43 | 0.72 | 18SQV | 1 | 1 | 1 | 4 | 6 | 1 | 4 |
| 18ST4 | 0.51 | 0.19 | 0.31 | 0.44 | 0.82 | 0.43 | 0.76 | 18ST4 | 1 | 1 | 4 | 6 | 7 | 1 | 5 |
| ACT | 1.18 | 0.73 | 0.44 | 0.66 | 0.53 | 1.05 | 1.11 | ACT | 8 | 7 | 5 | 8 | 4 | 8 | 9 |
| HPRT | 0.67 | 0.39 | 0.27 | 0.27 | 0.43 | 0.71 | 0.64 | HPRT | 3 | 3 | 3 | 1 | 1 | 5 | 3 |
| PBGD | 1.10 | 0.66 | 0.53 | 0.76 | 0.98 | 1.12 | 1.03 | PBGD | 7 | 6 | 6 | 9 | 9 | 9 | 8 |
| G6PD | 0.85 | 0.49 | 0.21 | 0.50 | 0.92 | 0.94 | 0.89 | G6PD | 4 | 4 | 1 | 7 | 8 | 7 | 7 |
| GAPDH | 1.26 | 0.95 | 0.83 | 0.41 | 0.44 | 0.57 | 0.47 | GAPDH | 9 | 9 | 9 | 5 | 3 | 3 | 1 |
| TBP | 0.93 | 0.84 | 0.72 | 0.27 | 0.73 | 0.81 | 0.79 | TBP | 5 | 8 | 8 | 1 | 5 | 6 | 6 |
| RasC | 0.97 | 0.58 | 0.62 | 0.31 | 0.43 | 0.64 | 0.47 | RasC | 6 | 5 | 7 | 3 | 1 | 4 | 1 |
| BestKeeper | | | | | | | | | | | | | | | |
| Gene | Coefficient of correlation | | | | | | | Ranking | | | | | | | |
|  | ASAC | log/ stat | log | HS | OS | EN | Neff |  | ASAC | log/stat | log | HS | OS | EN | Neff |
| 18SQV | 0.57 | 0.84 | 0.99 | 0.44 | 0.24 | 0.31 | 0.46 | 18SQV | 8 | 1 | 1 | 8 | 8 | 6 | 6 |
| 18ST4 | 0.10 | 0.70 | 0.98 | 0.34 | 0.40 | 0.14 | 0.17 | 18ST4 | 9 | 5 | 2 | 9 | 7 | 8 | 8 |
| ACT | 0.69 | 0.70 | 0.77 | 0.96 | 0.70 | 0.83 | 0.89 | ACT | 5 | 4 | 5 | 1 | 4 | 2 | 1 |
| HPRT | 0.70 | 0.84 | 0.73 | 0.92 | 0.77 | 0.68 | 0.74 | HPRT | 4 | 2 | 6 | 3 | 3 | 4 | 3 |
| PBGD | 0.65 | 0.69 | 0.04 | 0.93 | 0.60 | 0.69 | 0.83 | PBGD | 6 | 6 | 9 | 2 | 5 | 3 | 2 |
| G6PD | 0.71 | 0.60 | 0.84 | 0.68 | 0.08 | 0.89 | 0.44 | G6PD | 3 | 7 | 4 | 6 | 9 | 1 | 7 |
| GAPDH | 0.77 | 0.49 | 0.47 | 0.51 | 0.95 | 0.04 | 0.59 | GAPDH | 2 | 9 | 7 | 7 | 1 | 9 | 5 |
| TBP | 0.65 | 0.82 | 0.43 | 0.83 | 0.53 | 0.19 | 0.14 | TBP | 7 | 3 | 8 | 5 | 6 | 7 | 9 |
| RasC | 0.78 | 0.51 | 0.92 | 0.87 | 0.79 | 0.36 | 0.62 | RasC | 1 | 8 | 3 | 4 | 2 | 5 | 4 |
| BestKeeper STD | | | | | | | | | | | | | | | |
| Gene | Standard deviation | | | | | | | Ranking | | | | | | | |
|  | ASAC | log/ stat | log | HS | OS | EN | Neff |  | ASAC | log/stat | log | HS | OS | EN | Neff |
| 18SQV | 0.30 | 0.16 | 0.21 | 0.10 | 0.20 | 0.20 | 0.34 | 18SQV | 2 | 1 | 1 | 1 | 2 | 1 | 2 |
| 18ST4 | 0.29 | 0.22 | 0.34 | 0.24 | 0.15 | 0.31 | 0.27 | 18ST4 | 1 | 2 | 3 | 2 | 1 | 3 | 1 |
| ACT | 1.20 | 0.51 | 0.57 | 1.13 | 0.65 | 1.07 | 1.31 | ACT | 8 | 4 | 7 | 8 | 5 | 8 | 9 |
| HPRT | 0.61 | 0.44 | 0.36 | 0.34 | 0.53 | 0.50 | 0.52 | HPRT | 3 | 3 | 4 | 4 | 4 | 4 | 4 |
| PBGD | 1.08 | 0.68 | 0.37 | 1.14 | 0.82 | 1.08 | 1.28 | PBGD | 7 | 5 | 5 | 9 | 9 | 9 | 8 |
| G6PD | 0.82 | 0.80 | 0.24 | 0.61 | 0.69 | 0.97 | 0.89 | G6PD | 4 | 6 | 2 | 7 | 6 | 7 | 7 |
| GAPDH | 1.51 | 1.12 | 0.56 | 0.36 | 0.75 | 0.27 | 0.55 | GAPDH | 9 | 9 | 6 | 5 | 8 | 2 | 5 |
| TBP | 0.93 | 0.99 | 0.70 | 0.37 | 0.30 | 0.67 | 0.46 | TBP | 5 | 7 | 8 | 6 | 3 | 6 | 3 |
| RasC | 1.02 | 1.00 | 0.81 | 0.32 | 0.74 | 0.50 | 0.65 | RasC | 6 | 8 | 9 | 3 | 7 | 5 | 6 |
| RefFinder | | | | | | | | | | | | | | | |
| Gene | Geomean of ranking values | | | | | | | Ranking | | | | | | | |
|  | ASAC | log/ stat | log | HS | OS | EN | Neff |  | ASAC | log/stat | log | HS | OS | EN | Neff |
| 18SQV | 1.86 | 2.06 | 1.86 | 2.78 | 3.22 | 1.19 | 3.76 | 18SQV | 3 | 2 | 2 | 3 | 3 | 1 | 5 |
| 18ST4 | 1.57 | 1.19 | 2.45 | 5.12 | 2.11 | 2.63 | 2.99 | 18ST4 | 1 | 1 | 3 | 6 | 2 | 3 | 4 |
| ACT | 8.74 | 5.05 | 5.44 | 8.00 | 5.38 | 8.24 | 8.00 | ACT | 9 | 6 | 6 | 8 | 7 | 8 | 8 |
| HPRT | 1.73 | 4.23 | 3.94 | 1.41 | 1.41 | 3.66 | 1.97 | HPRT | 2 | 4 | 4 | 1 | 1 | 4 | 2 |
| PBGD | 7.74 | 4.95 | 5.42 | 9.00 | 8.74 | 8.74 | 9.00 | PBGD | 8 | 5 | 5 | 9 | 9 | 9 | 9 |
| G6PD | 4.47 | 2.34 | 1.19 | 5.18 | 7.67 | 6.74 | 7.00 | G6PD | 4 | 3 | 1 | 7 | 8 | 7 | 7 |
| GAPDH | 7.45 | 9.00 | 8.74 | 3.83 | 4.43 | 1.86 | 1.86 | GAPDH | 7 | 9 | 9 | 4 | 5 | 2 | 1 |
| TBP | 5.73 | 7.00 | 6.96 | 5.01 | 5.01 | 6.24 | 5.42 | TBP | 6 | 7 | 7 | 5 | 6 | 6 | 6 |
| RasC | 4.68 | 8.00 | 7.97 | 2.15 | 3.96 | 4.73 | 2.71 | RasC | 5 | 8 | 8 | 2 | 4 | 5 | 3 |

**Supplementary Table S2**. Calculated fold-changes of expression of target genes (CSP21, HSP90) based on normalization with a combination of the two best ranked RGs assessed by all algorithms and a comprehensive ranking and all RGs independently. (CR; comprehensive ranking. NF; NormFinder. GN; geNorm. BK; BestKeeper coefficient of correlation. BKSTD; BestKeeper Standard Deviation. RF; refFinder.)

| EN | | | OS | | | HS | | |
| --- | --- | --- | --- | --- | --- | --- | --- | --- |
| Algorithm | best two RGs | fold change | Algorithm | best two RGs | fold change | Algorithm | best two RGs | fold change |
| CR/NF/GN/ BKSTD/RF | 18SQV/GAPDH | 370.4 | CR/NF/RF | HPRT/18ST4 | 20.5 | CR/RF | HPRT/RasC | 30.3 |
| BK | G6PD/ACT | 329.3 | GN | HPRT/RasC | 16.2 | GN | HPRT/TBP | 27.1 |
|  |  |  | BK | GAPDH/RasC | 13.7 | NF | G6PD/HPRT | 22.1 |
|  |  |  | BKSTD | 18ST4/TBP | 27.7 | BK | ACT/PBGD | 39.0 |
|  |  |  |  |  |  | BKSTD | 18SQV/RasC | 28.7 |
| RG |  | fold change | RG |  | fold change | RG |  | fold change |
| CSP21 |  | **364.0** | HSP90 |  | **22.4** | HSP90 |  | **25.8** |
| 18SQV |  | 279.6 | 18SQV |  | 22.8 | 18QV |  | 23.9 |
| 18ST4 |  | 441.6 | 18ST4 |  | 24.2 | 18ST4 |  | 33.0 |
| ACT |  | 124.9 | ACT |  | 10.5 | ACT |  | 36.6 |
| HPRT |  | 186.3 | HPRT |  | 16.9 | HPRT |  | 27.1 |
| PBGD |  | 252.0 | PBGD |  | 13.4 | PBGD |  | 41.4 |
| G6PD |  | 552.1 | G6PD |  | 11.2 | G6PD |  | 17.1 |
| GAPDH |  | 410.2 | GAPDH |  | 12.0 | GAPDH |  | 24.7 |
| TBP |  | 928.8 | TBP |  | 32.1 | TBP |  | 27.1 |
| RasC |  | 621.3 | RasC |  | 15.4 | RasC |  | 33.5 |
